# Multicenter retrospective cohort study of the sequential use of the antibody-drug conjugates (ADCs) trastuzumab deruxtecan (T-DXd) and sacituzumab govitecan (SG) in patients with HER2-low metastatic breast cancer (MBC)

**DOI:** 10.1038/s41523-025-00748-5

**Published:** 2025-04-15

**Authors:** Laura A. Huppert, Reshma Mahtani, Samantha Fisch, Naomi Dempsey, Sarah Premji, Angelina Raimonde, Saya Jacob, Laura Quintal, Michelle Melisko, Jo Chien, Ana Sandoval, Lauren Carcas, Manmeet Ahluwalia, Natasha Harpalani, Jenna Hoppenworth, Anne Blaes, Kelly Blum, Mi-Ok Kim, Dame Idossa, Ruta Rao, Karthik V. Giridhar, Hope S. Rugo

**Affiliations:** 1 University of California, San Francisco, San Francisco, CA USA; 2 Miami Cancer Institute, Baptist HealthSouth Florida, Miami, FL USA; 3 Mayo Clinic, Rochester, Rochester, MN USA; 4 Rush, Chicago, IL USA; 5 University of Minnesota, Minneapolis, MN USA

**Keywords:** Breast cancer, Breast cancer

## Abstract

Antibody drug conjugates (ADCs) have improved outcomes for patients with metastatic breast cancer (MBC), but there is little data about the sequential use of these agents. In this multicenter retrospective cohort study, we identified 84 patients with HER2-low MBC treated sequentially with trastuzumab deruxtecan (T-DXd) and sacituzumab govitecan (SG) in either order at 5 institutions between 2020–2024. We evaluated clinical parameters associated with time to treatment failure (TTF) and real-world overall survival (rwOS). Median TTF was longer for ADC1 than ADC2, irrespective of HR-status, ADC sequence order, age ≤65 or >65 years, presence of visceral disease, or use of an intervening therapy. Younger age, longer time from MBC diagnosis to start of ADC1, and receipt of SG as ADC1 were associated with longer rwOS from start of ADC1. This cohort represents one of the first multicenter retrospective series of patients treated with sequential ADCs for HER2-low MBC, which may inform clinical practice.

## Introduction

Antibody drug conjugates (ADCs) are an expanding class of biotherapeutics that have improved the therapeutic index of systemic chemotherapy. ADCs have demonstrated statistically significant improvements in progression free survival (PFS) and overall survival (OS) compared to chemotherapy of physician’s choice in several phase III clinical trials including for patients with metastatic triple negative breast cancer (mTNBC), HR + /HER2- metastatic breast cancer (MBC), and in HER2-low MBC^1–4^. Based on these studies, three ADCs are now FDA-approved for the treatment of HER2-negative MBC: sacituzumab govitecan (SG) for mTNBC^1^ and HR + /HER2- MBC^2^, trastuzumab deruxtecan (T-DXd) for HER2-low MBC^3^, and most recently Dato-DXd for HR + /HER2- MBC^4^. These ADCs have different antibody targets (TROP2 for SG and Dato-DXd and HER2 for T-DXd); all utilize topoisomerase I (TOP-1) inhibitor payloads. Additional ADCs are in development with multiple ongoing phase III trials in both early-stage and metastatic disease.

Given concurrent clinical trial development and the relevant phase III clinical trials excluded the use of prior ADCs, little is known about optimal ADC sequencing and whether resistance develops secondary to the target or the payload. In HER2 positive MBC, data suggests sequential use of HER2-targeted ADCs can be effective^5,6^. DESTINY-Breast02 randomized patients with HER2 + MBC previously treated with T-DM1 to receive T-DXd vs. trastuzumab or lapatinib with capecitabine and demonstrated a remarkable improvement in PFS (T-DXd 17.8 months versus TPC 6.9 months [HR 0.36 *p* < 0.0001]) and OS^6^. However, prospective data to guide the sequential use of T-DXd and SG in patients with HER2-negative MBC are lacking.

Several outstanding questions impact decisions about the use of available ADCs and real-world data can provide important information to help optimize treatment sequencing until prospective data become available. First, it is important to understand the safety and efficacy of these agents in a real-world population with diverse patient characteristics, including patients treated in later line settings and with multiple comorbidities who were likely ineligible for the phase III trials that lead to regulatory approval. Second, sequential use of ADCs may impact safety and efficacy outcomes, requiring further characterization. Third, it is essential to identify biomarkers that can help clarify mechanisms of response and resistance to ADCs to inform future sequencing and treatment strategies.

Here, we report one of the first multi-center retrospective case series of patients treated with the sequential use of T-DXd and SG, in either order, for HER2-low MBC. We describe patient characteristics and evaluate time to treatment failure (TTF), real-world overall survival (rwOS), and safety data, providing key data about the safety and efficacy of sequential use of ADCs.

## Results

### Patient demographic and tumor characteristics prior to ADC1

84 patients with HER2-low MBC were identified and included in this analysis. Patient demographic and disease characteristics by HR status are shown in Table 1. Fifty-six patients had HR + /HER2-low MBC (66.7%) and 28 patients had HR-/HER2-low MBC (33.3%). Most patients were female (*n* = 83; 98.8%), non-Hispanic (*n* = 66, 78.6%), and white (*n* = 62, 73.8%). The median age at start of ADC1 was 60.4 years (range 23.0–81.7 years) for patients with HR + /HER2-low disease and 54.0 years (range 37.5–79.1 years) for patients with HR-/HER2-low disease. Most patients had tumors with ductal histology (*n* = 64, 76.2%), with a small number of patients who had lobular or mixed ductal/lobular histology (*n* = 9 and *n* = 6 respectively, 17.9% combined). Nineteen patients (22.6%) had de novo metastatic disease. Most patients had visceral disease (*n* = 65, 77.4%) and a minority of patients had central nervous system (CNS) metastases (*n* = 14, 16.7%) prior to ADC1.

**Table 1:** Demographic data and treatment history

	HR + /HER2-low MBC (*n* = 56)	HR-/HER2-low MBC (*n* = 28)
**Demographic Data**		
Median age at start of ADC1, yrs (range)	60.4 (23.0–81.7)	54.0 (37.5–79.1)
Sex, n (%)		
Female	55 (98.2%)	28 (100.0%)
Male	1 (1.8%)	0 (0%)
Ethnicity, n (%)		
Hispanic	8 (14.3%)	9 (32.1%)
Non-Hispanic	47 (83.9%)	19 (67.9%)
Unknown	1 (1.8%)	0 (0%)
Race, n (%)		
Asian	4 (7.1%)	3 (10.7%)
Black	3 (5.4%)	5 (17.9%)
White	44 (78.6%)	18 (64.3%)
Other/unknown	5 (8.9%)	2 (7.1%)
Histology, n (%)		
Ductal	41 (73.2%)	23 (82.1%)
Lobular	7 (12.5%)	2 (7.1%)
Mixed ductal/lobular	5 (8.9%)	1 (3.6%)
Other/unknown	3 (5.4%)	2 (7.1%)
De novo metastatic disease, n (%)	12 (21.4%)	7 (25.0%)
Sites of metastatic disease prior to ADC1		
Bone	41 (73.2%)	20 (71.4%)
Liver	34 (60.7%)	11 (39.3%)
Lung	20 (35.7%)	14 (50.0%)
CNS	8 (14.3%)	6 (21.4%)
Visceral disease prior to start of ADC1	47 (83.9%)	18 (64.3%)
**Treatment History**		
Median time from MBC diagnosis to start of ADC1, months (range)	44.0 (0.7–199.3)	10.2 (0.5–59.6)
Median lines of therapy prior to ADC1 by type of therapy:		
Medan lines ET, number (range)	2 (0–6)	0 (0–1)
Median lines chemotherapy, number (range)	2 (0–7)	1 (0–4)
Median total lines of therapy, number (range)	4 (0–10)	2 (0–5)
Prior CDK 4/6 inhibitor use, n (%)	45 (80.4%)	n/a
Median time on ET for MBC, months (range)	30.6 (0–145.0)	n/a
Prior immunotherapy, n (%)	13 (23.2%)	18 (64.3%)

*HR* hormone receptor, *MBC* metastatic breast cancer, *ET* endocrine therapy, *CDK* cyclin dependent kinase.

### Systemic therapy in the metastatic setting prior to ADC1

Systemic therapy in the metastatic setting prior to ADC1 is shown in Table 1. For patients with HR + /HER2-low MBC (*n* = 56), median time from MBC diagnosis to ADC1 was 44.0 months (range 0.7–199.3 months). Median prior lines of therapy for MBC prior to ADC1 were 4 (range 0–10), including a median of 2 prior lines of endocrine therapy (range 0–6) and 2 prior lines of chemotherapy (range 0–7). Most patients received prior CDK4/6 inhibitor therapy (*n* = 45, 80.4%) and median time on endocrine therapy for MBC was 30.6 months (range 0–145.0 months). A minority of patients with HR + /HER2-low disease received prior immunotherapy for MBC (*n* = 13, 23.2%) in the context of a clinical trial.

For patients with HR-/HER2-low MBC (*n* = 28), median time from MBC diagnosis to ADC1 was 10.2 months (range 0.5–59.6 months). Median prior lines of therapy for MBC prior to ADC1 were 2 (including chemotherapy, PARP inhibitors, range 0–5), including a median of one prior line of chemotherapy (range 0–4). More than half of the patients received prior immunotherapy for MBC (*n* = 18, 64.3%).

### Efficacy data and survival status by HR status and ADC sequence order

**Fig. 1:**
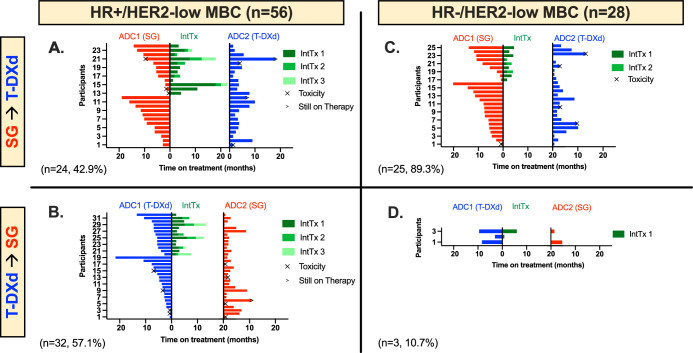
Time to treatment failure (TTF) for ADC1 and ADC2 with or without intervening therapy (IntTx). **A–D** Double-headed Swimmer Plots demonstrating time on treatment in months for each antibody drug conjugates (ADC). Patients with HR + /HER2-low MBC are shown at left (**A**, **B**) and those with HR-/HER2-low MBC are shown at right (**C**, **D**). Patients receiving sacituzumab govitecan (SG, red) followed by trastuzumab deruxtecan (T-DXd, blue) are shown in the top row (**A**, **C**) and patients receiving T-DXd followed by SG are in the bottom row (**B**, **D**). For each tornado plot, each bar represents an individual patient. The time on treatment for the first ADC (ADC1) is shown at left, if the patient received an intervening therapy (InTx) the time on treatment is shown in green in the middle (with different shades of green representing subsequent lines of intervening therapy; if no green bar then the patient did not receive an intervening therapy), and the time on treatment for the second ADC (ADC2) is shown at right. Patients stopped ADC therapy due to progression unless otherwise noted as due to toxicity (X) or if they were still on therapy at the time of data cutoff (>).

Efficacy data and survival status by HR status and ADC sequence order is summarized in Fig. 1 and Table 2 (also see Supplementary Fig. 1 for individual rwOS Kaplan Meier curves by subtype and ADC sequence order).

**Table 2:** Efficacy and survival status by hormone receptor status and ADC sequence order

	HR + /HER2-low MBC (*n* = 56)		HR-/HER2-low MBC (*n* = 28)	
ADC sequence order	SG → TDXd 24 (42.9%)	TDXd → SG 32 (57.1%)	SG → TDXd 25 (89.3%)	TDXd → SG 3 (10.7%)
Median total lines of therapy prior for MBC prior to ADC1, number (range)	3.0 (0–9)	4.5 (2–10)	2.0 (0–5)	3.0 (1–5)
Median lines of chemotherapy for MBC prior to ADC1, number (range)	2.0 (0–7)	2.0 (0–6)	1.0 (0–4)	2.0 (0–3)
Use of intervening therapy between ADCs, n (%)	12 (50.0%)	13 (40.6%)	9 (36.0%)	2 (66.7%)
Real-world response rate by investigator assessment, n (%)				
ADC1	17/22 (77.3%)	15/32 (46.9%)	17/25 (68.0%)	1/3 (33.3%)
ADC2^a^	8/23 (34.8%)	5/29 (17.2%)	7/21 (33.3%)	0/2 (0.0%)
Median TTF, mo				
ADC1	6.3	5.3	7.5	Undetermined^b^
ADC2	3.6	2.1	2.8	Undetermined^b^
Median rwOS, mo				
ADC1	22.8	17.7	16.5	Undetermined^b^
ADC2	7.8	5.8	6.5	Undetermined^b^

*ADC* antibody drug conjugate, HR hormone receptor, *MBC* metastatic breast cancer, *SG* sacituzumab govitecan, *T-DXd* trastuzumab deruxtecan, *ADC1* first antibody drug conjugate, *ADC2* second antibody drug conjugate, *TTF* time to treatment failure, *rwOS* real world overall survival.

^a^Several responses for ADC2 are not evaluable given patients discontinued therapy prior to first scan, hence change in denominator.

^b^Undetermined due to small sample size.

Of the patients with HR + /HER2-low MBC (*n* = 56), 24 patients (42.9%) were treated with SG prior to T-DXd (Fig. 1A). These patients had a median of 3 prior lines of total therapy for MBC prior to SG (ADC1) (range 0–9), including 2 prior lines of chemotherapy (range 0–7). Half of the patients (*n* = 12, 50.0%) received an intervening therapy between ADCs. The real-world response rate for evaluable patients was 17/22 (77.3%) for SG (ADC1) and 8/23 (34.8%) for T-DXd (ADC2). The median TTF was 6.3 months for SG (ADC1) and 3.6 months for T-DXd (ADC2). The median rwOS from the start of ADC1 was 22.8 months for SG (ADC1) and 7.8 months from the start of ADC2 for T-DXd (ADC2).

Of the patients with HR + /HER2-low MBC (*n* = 56), 32 patients (57.1%) were treated with T-DXd prior to SG (Fig. 1B). These patients had a median of 4.5 prior lines of total therapy for MBC prior to T-DXd (ADC1) (range 2–10), including 2 prior lines of chemotherapy (range 0–6). Thirteen patients (40.6%) received an intervening therapy between ADCs. The real-world response rate was 15/32 (46.9%) for T-DXd (ADC1) and 5/29 (17.2%) for SG (ADC2). The median TTF was 5.3 months for T-DXd (ADC1) and 2.1 months for SG (ADC2). The median rwOS was 17.7 months from the start of ADC1 for T-DXd (ADC1) and 5.8 months from the start of ADC2 for SG (ADC2).

Of the patients with HR-/HER2-low MBC (*n* = 28), 25 patients (89.3%) were treated with SG prior to T-DXd (Fig. 1C). These patients had a median of 2 prior lines of total therapy for MBC prior to SG (ADC1) (range 0–5), including 1 prior line of chemotherapy (range 0–4). Nine patients (36.0%) received an intervening therapy between ADCs. The real-world response rate was 17/25 (68.0%) for SG (ADC1) and 7/21 (33.3%) for T-DXd (ADC2). The median TTF was 7.5 months for SG (ADC1) and 2.8 months for T-DXd (ADC2). The median rwOS was 16.5 months for SG (ADC1) and 6.5 months for T-DXd (ADC2).

Of the patients with HR-/HER2-low MBC, 3 patients (10.7%) were treated with T-DXd prior to SG (Fig. 1D). These patients had a median of 3 prior lines of total therapy for MBC prior to T-DXd (ADC1) (range 1–5), including 2 prior lines of chemotherapy (range 0–3). Two patients (66.7%) received an intervening therapy between ADCs. The real-world response rate was 1/3 (33.3%) for T-DXd (ADC1) and 0/2 (0.0%) for SG (ADC2). The median TTF and rwOS were undetermined due to small sample size.

Across HR-status and ADC sequence order, most patients had longer TTF with ADC1 vs. ADC2 (*n* = 65, 77.4%), but exceptions existed and some patients had a longer TTF with ADC2 vs. ADC1 (*n* = 19, 22.6%) (Fig. 1). For the latter group with longer TTF for ADC2, median TTF for ADC1 and ADC2 were 2.5 months and 6.9 months, respectively. Within this subset of patients, the majority discontinued ADC1 due to progression (*n* = 13, 68.4%) with a smaller number discontinuing for toxicity (*n* = 6, 31.6%), half of which were due to an ILD diagnosis while on T-DXd (*n* = 3).

### Efficacy data and survival status by key clinical parameters

TTF and rwOS was evaluated by key clinically relevant subgroups, including age ≤ 65 years vs. >65 years, presence of visceral disease prior to ADC1, presence of CNS metastases prior to ADC1, and de novo vs. non-de novo MBC although these analyses are limited by small numbers (Supplementary Fig. 2). Among patients with HR + /HER2-low MBC treated with ADCs in either order, there was no difference in median TTF of ADC1 or ADC2 in any of these subgroups (Supplementary Fig. 2A, B). When analyzing rwOS from start of ADC1, there were no significant differences in rwOS between the subgroups for either ADC sequence order except for patients who received SG prior to T-DXd by age: patients age ≤65 years (n = 17) had a longer rwOS compared to patients age >65 years (n = 7) (26.0 vs. 15.7, p = 0.05) (Supplementary Fig. 2A, B).

Among patients with HR-/HER2-low MBC who were treated with SG prior to T-DXd (*n* = 25), there was no difference in TTF of ADC1 and ADC2 by age ( ≤ 65 years vs. >65 years), de novo vs. non-de novo MBC, or presence of visceral metastases. Patients without CNS disease (*n* = 19) had a longer TTF of ADC1 (7.9 vs 5.2 months, *p* = 0.047) and rwOS ADC1 (19.3 vs 11.6 months, *p* < 0.0001) vs. patients with CNS disease (*n* = 6) (Supplementary Fig. 2C). Subgroup analyses for patients with HR-/HER2-low treated with T-DXd prior to SG were not possible due to the small sample size (*n* = 3).

### Time to treatment failure (TTF) with and without intervening therapies

Next, we evaluated whether the use of intervening therapies between ADCs affected outcomes. Twenty-five patients with HR + /HER2- disease (44.6%) and 11 patients with HR-/HER2-low MBC (39.3%) received one or more intervening therapies between ADCs. The most common intervening therapies included: eribulin (*n* = 13, 36.1%), liposomal doxorubicin (*n* = 10, 27.8%), gemcitabine and carboplatin (*n* = 9, 25.0%), capecitabine (*n* = 6, 16.7%), paclitaxel (*n* = 6, 16.7%), vinorelbine (*n* = 4, 11.1%), olaparib (*n* = 3, 8.3%), and fulvestrant (*n* = 3, 8.3%). Adjusting for HR status, there was no statistically significant difference in TTF of ADC2 in patients who received an intervening therapy vs not (HR 1.497, 95% CI 0.921–2.434, *p* = 0.104), and there was no statistically significant difference in time on treatment with the first intervening therapy vs. ADC2 in patients who did not receive an intervening therapy (HR 0.978 (0.618, 1.547), *p* = 0.923).

### Factors associated with rwOS from start of ADC1 in univariable and multivariable analysis

We then evaluated factors associated with rwOS from the start of ADC1 in univariate and multivariate analysis stratified by HR status, as shown in Table 3 including the following variables: age at start of ADC1, time from MBC diagnosis to start of ADC1, lines of chemotherapy prior to ADC1, receipt of intervening therapies between ADCs, ADC treatment order (SG to T-DXd vs. T-DXd to SG), presence of visceral metastases, presence of CNS metastases, de novo MBC, ADC1 dose reduction, and ADC2 dose reduction. In univariate analysis, younger age at start of ADC1 (HR 0.829, 95% 0.730–0.943 using linear terms; HR 1.002, 95% CI 1.001–1.003 using quadradic terms, *p* = 0.030) and longer time from MBC diagnosis to start of ADC1 (HR 0.988, 95% CI 0.978–0.997, *p* = 0.011) were associated with longer rwOS from start of ADC1. In multivariate analysis, age at start of ADC1 (HR 0.864, 95% CI 0.746–1.000 using linear terms, *p* = 0.050), time from MBC diagnosis to start of ADC1 (HR 0.985, 95% CI 0.974–0.997, *p* = 0.012), and ADC sequence order SG→T-DXd vs. T-DXd to SG (HR 0.461, 95% CI 0.246–0.864, *p* = 0.016) were associated with longer rwOS from start of ADC1.

**Table 3:** Factors associated with real-world overall survival from start of ADC1 in univariable and multivariable analysis

	Simple (or unadjusted effects) *N* = 84		Multivariable (or adjusted effects) *N* = 84	
Stratified Cox analysis by hormone receptor status	HR (95% CI)	*p* value	HR (95% CI)	*p* value
Age (yrs) at ADC1^1^	0.83 (0.73, 0.94)	0.030*	0.86 (0.75, 1.00)	0.050*
Age (yrs) at ADC1^2^	1.00 (1.00, 1.00)		1.00 (1.00, 1.00)	0.078
Months MBC to ADC1	0.99 (0.98, 0.997)	0.011*	0.99 (0.97, 0.997)	0.012*
Lines of chemotherapy prior to ADC1	1.06 (0.87, 1.28)	0.570	1.22 (0.97, 1.53)	0.087
Receipt of intervening therapy	0.78 (0.46, 1.32)	0.357	NA	NA
SG → T-DXd vs T-DXd → SG	0.57 (0.32, 1.02)	0.058	0.46 (0.25, 0.86)	0.016*
Visceral metastasis	1.25 (0.66, 2.36)	0.500	1.25 (0.64, 2.44)	0.507
CNS metastasis	1.55 (0.81, 2.99)	0.189	NA	NA
De novo MBC	0.72 (0.37, 1.37)	0.311	NA	NA
ADC1 dose reduction	0.61 (0.35, 1.07)	0.082	NA	NA
ADC2 dose reduction	1.20 (0.68, 2.10)	0.531	NA	NA

*P* values of ≤0.05 was considered statistically significant and indicated with a bold and (*).

*HR* hazard ratio, *CI* confidence interval, *ADC* antibody drug conjugate, *yrs* years, *MBC* metastatic breast cancer, *SG* sacituzumab govitecan, *T-DXd* trastuzumab deruxtecan, *CNS* central nervous system.

### Safety data by hormone receptor status and ADC sequence order

Safety data by hormone receptor status and ADC sequence order is summarized in Table 4. During treatment with SG, 41/84 patients (48.8%) required a dose reduction due to lab abnormalities (*n* = 26, 31.0%), symptoms (*n* = 12, 14.3%), or other reasons (*n* = 3, 3.6%). There was no statistically significant difference in rates of dose reduction by HR-status, whether SG was given as ADC1 vs. ADC2, presence or absence of visceral disease, or age ≤65 years or > 65 years. Eight patients (9.5%) discontinued SG due to toxicity per investigator assessment, including 7 patients with HR + /HER2-low MBC (12.5%) and one patient with HR-/HER2-low MBC (3.6%). Most patients received growth factor support during treatment with SG: 35/56 patients with HR + /HER2-low MBC (62.5%) and 18/28 patients with HR-/HER2-low MBC (64.3%). Of those who received growth factor support, about half received therapy as primary prophylaxis (20/35 [57.1%] HR + /HER2-low; 10/18 [55.6%] HR-/HER2-low), and half as secondary prophylaxis for neutropenia (15/35 [42.9%] HR + /HER2-low; 8/18 [44.4%] HR-/HER2-low). 16/84 total patients (19.0%) required a treatment delay due to neutropenia.

**Table 4:** Safety data by hormone receptor status and ADC sequence order

	HR + /HER2-low MBC (*n* = 56)		HR-/HER2-low MBC (*n* = 28)	
ADC sequence order	SG → TDXd	TDXd → SG	SG → TDXd	TDXd → SG
**During treatment with SG**	**SG as ADC1** (*n* = 24)	**SG as ADC2** (*n* = 32)	**SG as ADC1** (*n* = 25)	**SG as ADC2** (*n* = 3)
Required SG dose reduction	10 (41.6%)	19 (59.4%)	11 (44.0%)	1 (33.3%)
Due to lab abnormalities	6 (25.0%)	12 (37.5%)	7 (28.0%)	1 (33.3%)
Due to symptoms	4 (16.7%)	4 (12.5%)	4 (16.0%)	0
Due to other reasons	0	3 (9.4%)	0	0
Discontinued SG due to toxicity	3 (12.5%)	4 (12.5%)	1 (4.0%)	0
Received growth factor support during SG	14 (58.3%)	21 (65.6%)	17 (68.0%)	1 (33.3%)
Primary prophylaxis	7 (29.2%)	13 (40.6%)	10 (40.0%)	0
Secondary prophylaxis	7 (29.2%)	8 (25.0%)	7 (28.0%)	1 (33.3%)
Treatment delay due to neutropenia	4 (16.7%)	6 (18.8%)	6 (24.0%)	0
**During treatment with T-DXd**	**T-DXd as ADC2** (*n* = 24)	**T-DXd as ADC1** (*n* = 32)	**T-DXd as ADC2** (*n* = 25)	**T-DXd as ADC1** (*n* = 3)
Required T-DXd dose reduction	4 (16.7%)	8 (25.0%)	3 (12.0%)	0
Due to lab abnormalities	0	2 (6.3%)	2 (8.0%)	0
Due to symptoms	2 (8.3%)	3 (9.4%)	0	0
Due to other reason(s)	2 (8.3%)	3 (9.4%)	1 (4.0%)	0
Discontinued T-DXd due to toxicity	2 (8.3%)	4 (12.5%)	4 (16.0%)	0
Received growth factor support during SG	3 (12.5%)	3 (9.4%)	2 (8.0%)	0
Primary prophylaxis	3 (12.5%)	2 (6.3%)	2 (8.0%)	0
Secondary prophylaxis	0	1 (3.1%)	0	0
Treatment delay due to neutropenia	0	2 (6.3%)	0	0
Diagnosed with any grade ILD/ pneumonitis	2 (8.3%)	7 (21.9%)	5 (20.0%)	0
Grade 1–2	0	7 (21.9%)	0	0
Grade 3–4	1 (4.2%)	0	3 (12.0%)	0
Grade 5	1 (4.2%)	0	2 (8.0%)	0

*HR* hormone receptor, *MBC* metastatic breast cancer, *ADC1* first antibody drug conjugate, *ADC2* second antibody drug conjugates, *SG* sacituzumab govitecan, *T-DXd* trastuzumab deruxtecan, *ILD* interstitial lung disease.

During treatment with T-DXd, 15/84 patients (17.9%) required a dose reduction due to lab abnormalities (*n* = 4, 4.8%), symptoms (*n* = 5, 6.0%), or other reasons (*n* = 6, 7.1%). Patients who were age >65 years were more likely to have a T-DXd dose reduction (9/22, 40.9%) compared to patients who were ≤65 years (6/62, 9.7%) (*p* < 0.01) as were those with visceral disease (15/65, 23.1%) vs. those without visceral disease (0/19, 0.0%) (*p* = 0.03). There was no statistically significant difference in rates of dose reduction by HR-status and whether T-DXd was given as ADC1 vs. ADC2. Ten patients (11.9%) discontinued T-DXd due to toxicity per investigator assessment. Eight patients (9.5%) received growth factor support during treatment with T-DXd. Fourteen of 84 patients (16.7%) were diagnosed with any grade interstitial lung disease (ILD)/pneumonitis, including 9 patients with HR + /HER2-low MBC (16.1%) and 5 patients with HR-/HER2-low MBC (17.9%). Of the patients who developed ILD/pneumonitis, median age was 64 years (range 49–80 years), median body mass index was 24.9 (range 19–41), 3/14 patients (21.4%) had a prior smoking history, and 9/14 patients (64.3%) had metastatic disease involving lung. Half of the patients received T-DXd as ADC1 (*n* = 7, 50.0%) and the other half as ADC2 (*n* = 7, 50.0%). Median time to onset of ILD was 3.8 months (range 0.3–22.9 months). Peak grade of ILD/pneumonitis by investigator assessment was: grade 1 (*n* = 3, 3.6%), grade 2 (*n* = 4, 4.8%), grade 3 (*n* = 1, 1.2%), grade 4 (*n* = 3, 3.6%), and grade 5 (*n* = 3, 3.6%). Of the 7 patients with grade ≥ 3 ILD, 2 had HR + /HER2-low MBC (28.6%) and 5 had HR-/HER2-low MBC (71.4%). After the diagnosis of ILD, all patients (*n* = 14, 100.0%) were treated with corticosteroids. 9/14 patients (64.3%) required hospitalization and pulmonary consultation was obtained in 9/14 patients (64.3%). Two patients with grade 1 ILD were rechallenged with T-DXd after imaging resolution of ground glass opacities (one after completion of steroid taper, one during steroid taper); neither patient re-developed ILD with T-DXd rechallenge.

## Discussion

This is one of the first multicenter retrospective cohorts of patients treated with sequential ADCs for HER2-low MBC. Median TTF was longer for ADC1 than ADC2 in all subgroups, regardless of HR-status, ADC sequence order, age ≤65 or >65 years, presence of visceral disease, or use of an intervening therapy. However, there were notable exceptions with some patients demonstrating a longer duration of response to ADC2 vs. ADC1 (most of whom discontinued ADC1 for progression, others for toxicity).

Patients with HR + /HER2-low disease initially treated with T-DXd in our cohort (*n* = 32) demonstrated a 5.3 month TTF, which is significantly shorter than the reported median PFS (10.1 months) in the HR+ cohort of patients in the Destiny-Breast04 trial^3^. However, patients in our dataset had a median of 4.5 total lines of prior therapy for MBC compared to 3 lines of prior therapy in Destiny-Breast-04. In patients who were initially treated with SG, the median TTF in both HR+ and HR- disease in this cohort was similar to the median PFS in TROPICS-02 and ASCENT respectively (HR + : TTF 6.3 months in our cohort vs. PFS 5.5 months in TROPICS-02; HR- TTF 7.5 months in our cohort vs. PFS 5.6 months in ASCENT)^1,2^. However, given that the endpoint TTF does not censor for toxicity while PFS does, caution should be taken in cross-study interpretation of these endpoints from a real-world cohort and randomized studies. Additionally, comparisons of real-world response rate between our study and the objective response rates in the respective phase 3 ADC trials should also be interpreted with caution, as patients were treated in different lines of therapy, and our study did not include RECIST assessments, making our evaluation of response not directly comparable to RECIST-defined responses.

Several other multi-center retrospective studies have also evaluated the efficacy of the sequential use of ADCs in MBC^7–12^, with similar overall findings including an overall longer duration of response with ADC1 compared to ADC2. For example, in a multicenter US cohort by Abelman et. al. that identified patients treated with two ADCs (including experimental ADCs) (*n* = 68), median time to progression on ADC1 was 161 days vs. 77 days with ADC2; among the smaller cohort of patients treated with SG and T-DXd in either order, the median PFS data was strikingly similar between their cohort and our cohort^7^. A single center study by Mai et. al. analyzed patients receiving SG and T-DXd for HER2-negative MBC (*n* = 85) and found that 64/85 patients (75.3%) had a longer PFS for ADC1 vs. ADC2, with more notable differences in PFS with SG given first vs. second compared to T-DXd given first vs. second^8^. A multicenter French cohort by Poumeaud et al. including patients treated with both SG and T-DXd for HER2-negative MBC (*n* = 179) also observed longer median PFS for the first ADC compared to the second ADC, with no impact on efficacy of ADC2 in those with use of an intervening therapy between ADCs, similar to our data^9^. Using data from the Flatiron Health registry, Tarantino et. al. evaluated real-world efficacy of T-DXd in patients who had received prior SG (*n* = 58) vs. those who had not received prior SG (*n* = 61), and noted shorter median real-world PFS (rwPFS) (3.4 vs. 5.7mo, *p* = 0.005) and rwOS (9.0 vs. 14.5mo, *p* < 0.001) in patients who had received prior SG vs. those who had not received prior SG, although patients who received prior SG had a median of 3.0 prior lines of therapy vs. 2.0 for those who had not received prior SG^10^. This group also reported median rwPFS with different treatment regimens administered after T-DXd in the Flatiron database, and observed that chemotherapy seemed to perform better than another Topo1 ADC^11^.

Duration of response ADC1 vs. ADC2, whether the use of an intervening therapy affects the efficacy and safety and ADC2, and whether a particular ADC sequence order is superior are all important clinical questions. In our study, most patients derived a longer duration of response from ADC1 vs. ADC2 (*n* = 65, 77.4%), but there were a subset of patients who had a longer response to ADC2 vs. ADC1 (*n* = 19, 22.6%). Analysis of patient characteristics with a longer duration of response to ADC2 is of significant interest but is limited by the small number of patients in this subset; future work in larger patient cohort should specifically evaluate the unique clinical and molecular features of this subset of patients. It is also of interest to know whether the use of an intervening chemotherapy between ADCs may impacts the safety and efficacy of ADC2. In our study, among patients who received an intervening chemotherapy between ADCs, TTF for the first intervening therapy after ADC1 was similar to TTF for ADC2 in patients who were treated immediately with ADC2. However, there may be potential selection bias in choice of ADC2 vs. chemotherapy as next line of therapy, so future prospective data is needed to better address this question. It is also important to consider how the use of an intervening therapy may impact safety, including dose modifications and discontinuations, of ADC2; the sample size is too small to evaluate this question in our dataset, but it should be investigated in future studies. Finally, regarding optimal ADC sequencing order, in our multivariate analysis we found that ADC sequence order SG→T-DXd vs. T-DXd to SG (HR 0.461, 95% CI 0.246–0.864, p = 0.016) was associated with longer rwOS from start of ADC1; however our study was not designed the analyze the efficacy of a particular ADC sequence order given the heterogeneity of this cohort, so this finding should be interpreted with caution, and future prospective randomized studies can better address this important question.

In addition to our analysis of sequential ADC efficacy, we also analyzed real-world safety and toxicity data in our cohort by HR-status and ADC sequence order. Most retrospective ADC sequencing studies published to date have not evaluated safety, so this represents a unique contribution of our study. During treatment with SG, 41/84 patients (48.8%) required a dose reduction of SG and 8/84 patients (9.5%) discontinued SG due to toxicity (compared to 22% dose reduction and 4.7% discontinuation of SG for patients with mTNBC in ASCENT^1^ and 33.0% dose reduction and 6.0% discontinuation of SG for patients with HR + /HER2- MBC in TROPICS-02^2^). During treatment with T-DXd, 15/84 patients (17.9%) required a dose reduction of T-DXd and 10/84 patients (11.9%) discontinued T-DXd due to toxicity (compared to 20.8% dose reduction and 15.1% discontinuation rate in Destiny-Breast-04^3^). Importantly, there was no difference in rates of dose reduction by ADC sequence order in patients with either HR+ or HR- disease. Of note, during treatment with T-DXd, 14/84 patients (16.7%) were diagnosed with any grade interstitial lung disease (ILD)/pneumonitis, which is slightly higher than the percentage of patients diagnosed with ILD in T-DXd clinical trials (e.g., Destiny-Breast-03 10.5%^6^, Destiny-Breast-04 12.1%^3^, pooled analysis of 9 studies with T-DXd 15.4%^13^). Unfortunately, there were three patients (3.6%) with grade 5 ILD events in this cohort, which is a higher rate of grade 5 events than what was seen in the T-DXd clinical trials (e.g., Destiny-Breast-03 0.0%^6^; Destiny-Breast-04 0.8%^3^); we were unable to definitively adjudicate these cases in this retrospective review. Interestingly, most patients who were diagnosed with higher grade ILD had mTNBC, perhaps at least partially related to the fact that most of these patients received T-DXd as ADC2 so they may have had worse functional status and disease burden during treatment with T-DXd. Of note, two patients in this cohort with grade 1 ILD underwent re-challenge with T-DXd without recurrence of ILD, a finding that has similarly been reported in a pooled analysis of T-DXd clinical trials^14^.

To help inform optimal ADC sequencing in the future, additional data is needed to clarify mechanisms of response and resistance to ADCs. Prior work has demonstrated that resistance can develop due to modifications of the target or payload^15^. In the real-world analysis of sequential ADCs by Abelman et. al., Tumor DNA sequencing data were available for 20 patients who had received therapy with more than one ADC. Mutations in genes associated with topoisomerase I, including *TOP1*, *TOP3A*, and *TOP3B*, were identified in seven patients who exhibited cross-resistance to the second ADC, which carried a payload that inhibited topoisomerase I activity^7^. We recently presented preliminary analysis of NGS findings from this cohort^16^, although this analysis was limited by the use of different NGS assays and timepoints of collection. Therefore, prospective work is needed to validate and further clarify mechanisms of ADC resistance. Prospective trials that include biomarker analyses are planned, including TRADE-DXd which evaluates T-DXd followed by Dato-DXd or vice versa (NCT06533826, aka Translational Breast Cancer Research Consortium TBCRC-064), SERIES which evaluates T-DXd after SG (NCT06263543), and ENCORE, a prospective registry study of sequential ADCs for HER2-negative MBC (NCT06774027, aka TBCRC-067).

In addition to SG and T-DXd, Dato-DXd was recently FDA-approved for HR + /HER2- MBC^4^. With numerous other ADCs also in development, the challenge of ADC sequencing is expected to become increasingly complex. Key questions remain regarding the optimal sequencing of ADC therapies, the expected benefit for patients previously treated with one ADC who are subsequently treatment with a second or third, and whether toxicity rates vary depending on the timing of ADC administration in the treatment sequence. Differences in antibody target and payload will create even more variability in understanding and addressing these questions. There are also ongoing studies investigating the use of ADCs for early-stage breast cancer as neoadjuvant or adjuvant treatment, so understanding how the use of ADCs in the early-stage may impact efficacy in the metastatic setting is also an unanswered clinical question. Prospective trials can provide the most robust data to inform sequencing strategies, but these studies take time, resources, and may not adequately reflect real-world patient populations. Therefore, ongoing real-world retrospective evaluation of ADC sequencing can also continue to provide important initial data that can help refine our understanding of these agents.

This study has several notable strengths. First, this study answers important clinical questions in a space where prospective data does not yet exist, providing key initial data to guide patients and providers. Second, we performed detailed chart abstraction to capture demographic, clinical, and treatment data, which allowed for a thorough description of efficacy, safety, and survival status in this cohort. Third, the safety analysis performed in this cohort is unique as other real-world studies with sequential ADCs for HER2-negative MBC have not analyzed safety data to our knowledge, so this provides an important addition to the literature.

This study also has several limitations due to its retrospective nature. First, patients received ADCs at different timepoints in their care and in either sequence order, so this study is not able to assess the superiority of ADCs or ADC treatment order. Second, retrospective chart reviews are limited by written details included in the clinical record, so it is possible that certain demographic features, toxicities, or aspects of treatment history were not captured if they were not well-documented in the electronic medical record; we also did not perform RECIST reads in this retrospective study, which limits response analysis. Third, analysis of subgroups is somewhat limited by the smaller number of patients; additional analyses are needed in a larger patient cohort. Fourth, full assessment of adverse events associated with each ADC is limited by the retrospective nature of this study; we tried to specifically select AEs of interest that could be best described based on retrospective chart review (e.g., growth factor use, dose reduction, discontinuation, rates of ILD), but we were unable to adequately describe rates of other clinically-important AEs with retrospective review (e.g., rates of alopecia, nausea, diarrhea, etc.). Finally, given that this was a retrospective study, we did not have access to tissue and blood samples drawn at standardized timepoints for correlative assays; future prospective clinical trials that include correlative endpoints will be particularly valuable.

In summary, this retrospective case series describes the real-world outcomes of patients with HER2-low MBC treated with the sequential use of SG and T-DXd. Optimal sequencing strategies for ADCs is an unmet clinical need. Prospective studies are needed to validate these findings and to identify biomarkers of ADC response, resistance, and toxicity, which may inform optimal sequencing strategies in the future.

## Methods

### Study design and objectives

This is a multicenter retrospective cohort study of patients with HER2-low MBC treated sequentially with T-DXd and SG in either order, with or without intervening therapies, at five institutions (University of California San Francisco, Miami Cancer Institute - Baptist Health South Florida, Mayo Clinic Rochester, Rush, University of Minnesota) between 2020 and 2024 (data cutoff January 2024).

### Participant Identification

Patients were identified at each site via search of the electronic medical record or pharmacy databases. Patients were 18 years of age or older and had HR+ or HR-, HER2-low MBC. HR status was determined by most recent estrogen receptor (ER) and progesterone receptor (PR) test prior to ADC1 and tumors were considered HR+ if ER and/or PR was ≥10% per local pathology assessment. HER2-low was defined as HER2 immunohistochemistry (IHC) 1+ or 2+ without gene amplification by florescence in situ hybridization / in situ hybridization (FISH/ISH) in any tumor sample, regardless of stage and per local pathology assessment. All patients previously received both SG and T-DXd for MBC, in either order, with or without intervening therapies (e.g., a chemotherapy or other therapy administered in between ADCs). Patients were excluded during initial screening if they were treated with either ADC in combination with other therapies (rather than as monotherapy), if they received another ADC prior to treatment with T-DXd and SG, and/or if they had HER2-positive breast cancer. After chart extraction, additional patients were excluded if there were insufficient clinical records to complete an adequate chart review.

### Chart abstraction

Detailed information about patient demographic characteristics, treatment history, lab values, clinical outcomes, and survival dates were obtained via manual chart abstraction.

### Efficacy analysis

The real-world response rate was defined as the percentage of patients who had responding disease at the time of the first scan ordered at the discretion of the treating physician and was per radiology/clinical investigator assessment; RECIST reads were not performed for this retrospective study. Time to treatment failure (TTF) was defined as the time from start of ADC to discontinuation for any reason, including progression of disease, treatment toxicity, or death. rwOS was defined as the date from start of each ADC to date of death or last known clinical follow up.

### Safety analysis

Key safety parameters were extracted per clinical chart review and review of pharmacy records at each institution. The attribution for discontinuation of each ADC was determined by the investigator from each site per chart review and clinical assessment. Data about dose modification, dose delays, and growth factor administration was collected from pharmacy records at each site. We also evaluated patients who had documented interstitial lung disease (ILD)/pneumonitis. For the ILD subgroup analysis, adverse events were coded and graded according to the National Cancer Institute Common Terminology Criteria for Adverse Events (version 5.0) definitions per investigator assessment.

### Statistics

Data were analyzed using Prism Software (GraphPad; San Diego, CA) and R version 4.2.2 (www.r-project.org). Descriptive statistics were used to summarize numeric responses as rate of events (%) and median (range). Comparisons between groups were made using the unpaired *t* test, two-sided Fisher’s exact test, or log-rank (Mantel-Cox) test as appropriate. Survival distributions were estimated by the Kaplan-Meier non-parametric method. Univariable and multivariable analyses of factors associated with overall survival from start of ADC1 were evaluated using the Cox proportional-hazards model. All variables with *p* value < 0.1 on univariable analysis were included in the final multivariable model. *P* values of ≤0.05 were considered statistically significant.

### Institutional review board statement

This research was approved by the Institutional Review Board at all participating institutions: University of California San Francisco; Miami Cancer Institute, Baptist Health; Mayo Clinic; Rush; University of Minnesota. Informed consent was not required per IRB given the retrospective, non-interventional nature of this research. Research was performed in accordance with the Declaration of Helsinki.

## Supplementary information

Huppert et al Supplementary figures 2.21.25

Supplemental Figure 1

Supplemental Figure 2

## Data Availability

The datasets generated during the current study are not publicly available to protect patient privacy but are available from the corresponding author on reasonable request.
