# Supplementary material for: Multicenter retrospective cohort study of the sequential use of the antibody-drug conjugates (ADCs) trastuzumab deruxtecan (T-DXd) and sacituzumab govitecan (SG) in patients with HER2-low metastatic breast cancer (MBC)
Source: NPJ Breast Cancer. 2025 Apr 15;11:34. doi: 10.1038/s41523-025-00748-5 (PMC12000457; doi:10.1038/s41523-025-00748-5)
Supplement: Supplementary file 1 — Huppert et al Supplementary figures 2.21.25 [file 41523_2025_748_MOESM1_ESM.docx]

**SUPPLEMENTAL FIGURES**

**Supplemental Figure 1:** Real-world overall survival of ADC1 and ADC2

(**A-H**): Kaplan-Meier curves showing real-world overall survival (rwOS) in months for each ADC: Sacituzumab govitecan (SG, red) and trastuzumab deruxtecan (T-DXd, blue). The rwOS Kaplan-Meier curves for the first ADC (ADC1) are in the left column and the second ADC (ADC2) are in the right column

**Supplemental Figure 2:** Subgroup analyses of TTF and rwOS by HR-status and ADC sequence order

(**A-C**): Bar graphs showing, from left to right, the median time to treatment failure (TTF) of ADC1, median TTF of ADC2, and the median real-world overall survival (rwOS) for ADC1 for each of the following patient cohorts: (**A**). HR+/HER2-low metastatic breast cancer (MBC) treated with sacituzumab govitecan (SG) prior to T-DXd (T-DXd) (n=24). (**B**). HR+/HER2-low MBC treated with T-DXd prior to SG (n=32). (**C**). HR-/HER2-low MBC treated with SG prior to T-DXd (n=25). The subgroups of patients in each of these cohorts are indicated by the colored bar graphs, with the legend at the top right. Subgroup analyses for patients with HR-/HER2-low treated with T-DXd prior to SG were possible due to the small sample size (n=3). P values of ≤0.05 was considered statistically significant and indicated with a (*) above the relevant subgroup pairing.
