# Supplementary figures and images for: Multicenter retrospective cohort study of the sequential use of the antibody-drug conjugates (ADCs) trastuzumab deruxtecan (T-DXd) and sacituzumab govitecan (SG) in patients with HER2-low metastatic breast cancer (MBC)
Source: NPJ Breast Cancer. 2025 Apr 15;11:34. doi: 10.1038/s41523-025-00748-5 (PMC12000457; doi:10.1038/s41523-025-00748-5)

## HR+/HER2-low MBC

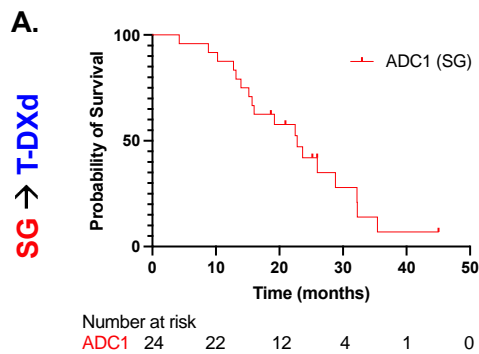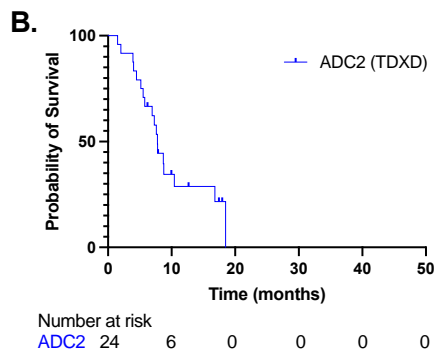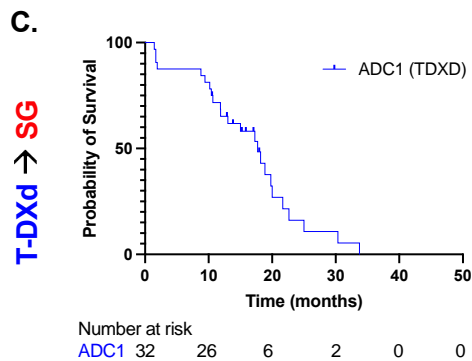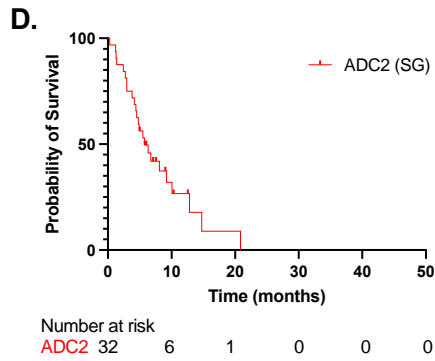

## HR-/HER2-low MBC

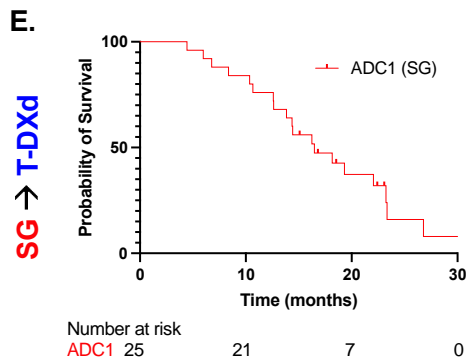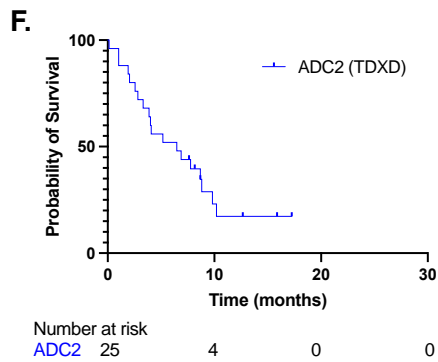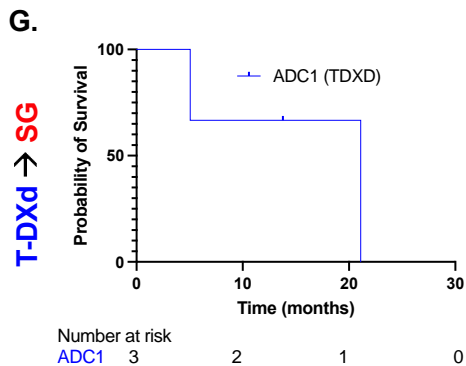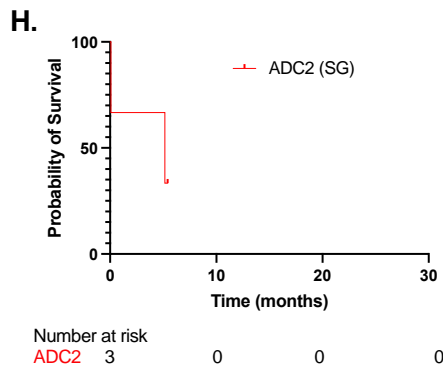

Supplement: Supplementary file 2 — Supplemental Figure 1 [file 41523_2025_748_MOESM2_ESM.pdf]

A.

HR+/HER2-low SG → T-DXd

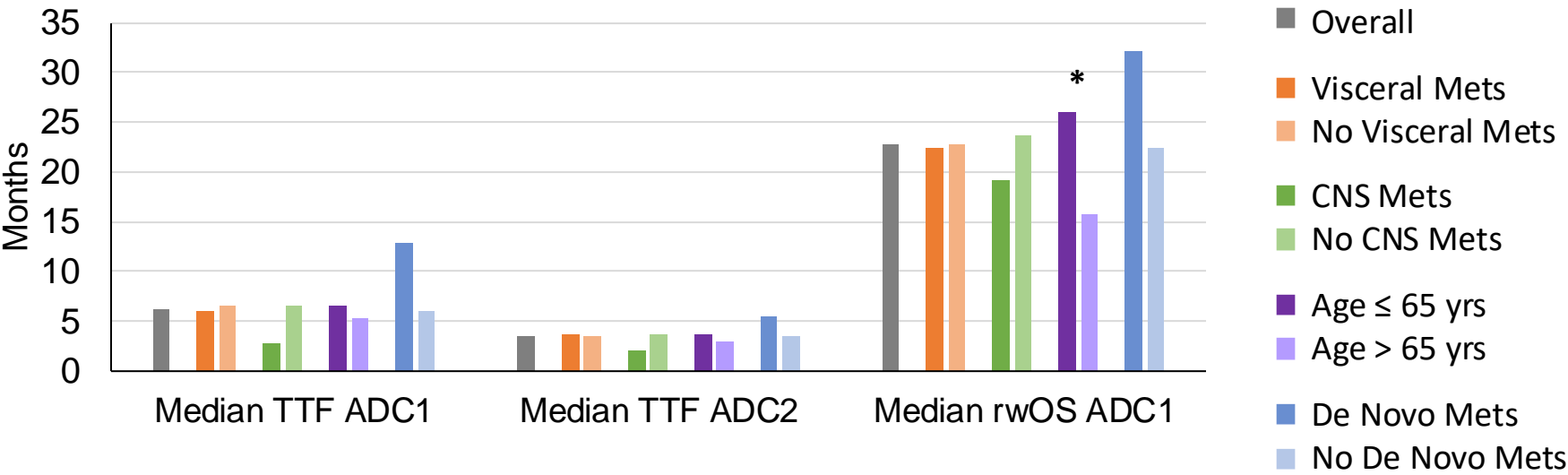

B.

HR+/HER2-low T-DXd → SG

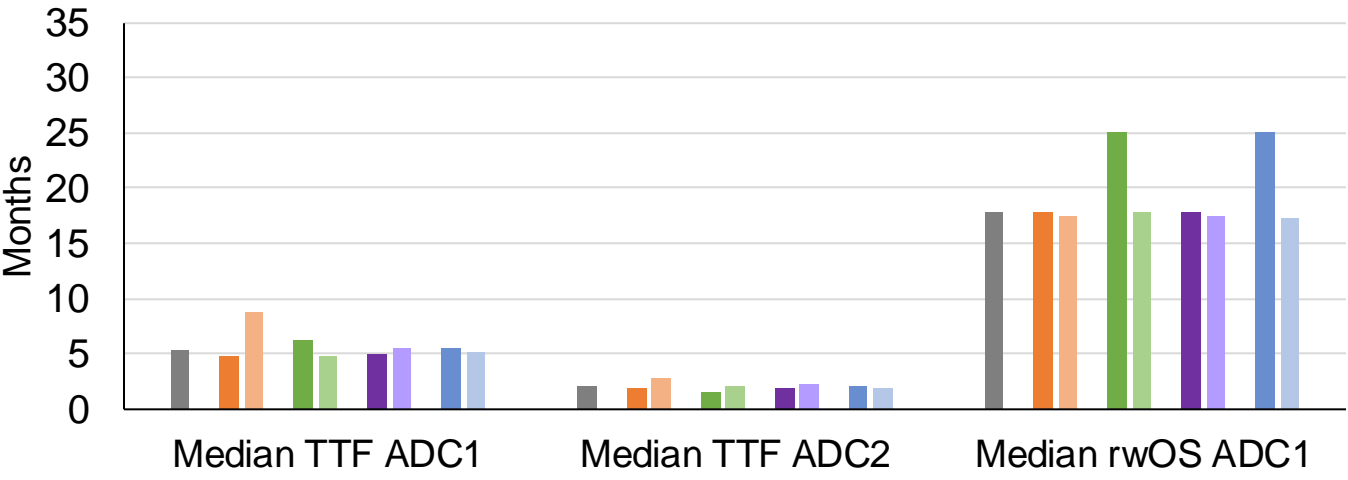

C.

HR-/HER2-low SG → T-DXd

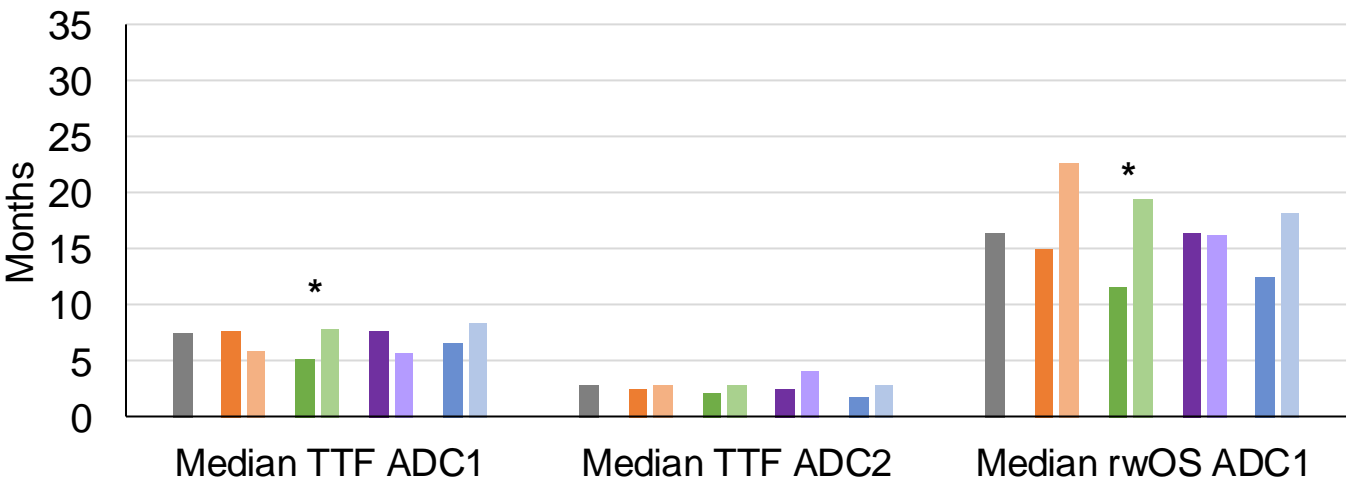

Supplement: Supplementary file 3 — Supplemental Figure 2 [file 41523_2025_748_MOESM3_ESM.pdf]
